# Wilson’s Herald of Health

**Published:** 1874-03

**Authors:** 


It is a matter of regret to see, in a journal called *Wilson's Herald of Health,* published at Atlanta, and edited by a once reputable physician, an advertisement of a "certain cure for consumption" inserted in the editorial columns.—*Medical and Surgical Reporter.*

